# Biomarcadores Endoteliais e Medicina Translacional: Ainda um Desafio

**DOI:** 10.36660/abc.20220533

**Published:** 2022-10-05

**Authors:** Renato Jorge Alves

**Affiliations:** 1 Irmandade da Santa Casa de Misericórdia de São Paulo Departamento de Medicina São Paulo SP Brasil Irmandade da Santa Casa de Misericórdia de São Paulo – Departamento de Medicina , São Paulo , SP – Brasil; 2 Faculdade de Ciências Médicas Santa Casa de São Paulo São Paulo SP Brasil Faculdade de Ciências Médicas da Santa Casa de São Paulo , São Paulo , SP – Brasil

**Keywords:** Aterosclerose, Estado Pré-Diabético, Resistência à Insulina, Diabetes Mellitus, Espessura Intima-Média Carotídea, Obesidade

O artigo “O Nível de Endocan Sérico pode ser Usado como Biomarcador para Prever Aterosclerose Subclínica em Pacientes Pré-Diabéticos?” ^1^ abordou um tema da maior relevância e originalidade, a investigação diagnóstica da aterosclerose subclínica em pacientes pré-diabéticos. Portanto, o estudo avaliou a concentração de endocan da molécula 1 específica da célula endotelial (ESM-1) em pacientes pré-diabéticos para verificar o papel dos níveis séricos de endocan na detecção de aterosclerose subclínica, auxiliado pela medição da camada espessura da íntima média carotidea (EIMC).

Devido à epidemia global de obesidade, há um número crescente de pacientes diagnosticados com diabetes tipo 2. Entretanto, antecedendo essa morbidade, está oculto um contingente ainda maior de indivíduos, aqueles em fase pré-diabética ou de resistência à insulina.

Sabe-se que a resistência à insulina aumenta o risco cardiovascular, pois está relacionada a um pior perfil lipídico, estado pró-inflamatório e disfunção endotelial. Ocorrem alterações endoteliais expressivas, aumentando a partir da expressão de marcadores inflamatórios. ^2^

Por outro lado, agregar ferramentas que contribuam para a detecção precoce desses biomarcadores nessa população é essencial para tentar mudar a evolução da doença. Entre eles, a medição do EIMC tornou-se um dos principais métodos de avaliação e diagnóstico. Além disso, além de ser um exame de fácil execução, correlaciona-se diretamente com alteração endotelial precoce na doença aterosclerótica. ^3 - 6^

O endocan, por sua vez, é um proteoglicano liberado pelas células endoteliais a partir de citocinas inflamatórias, que regularia o processo inflamatório. Está intimamente ligado à lesão endotelial. Foi demonstrado que os níveis séricos de endocan seriam maiores em pacientes diabéticos e com síndrome coronariana aguda. ^7 , 8^

No entanto, resultados intrigantes indicaram que os níveis séricos de endocan estariam baixos ou inalterados em pacientes pré-diabéticos, ^9 , 10^ em contraste com os valores de EIMC, que seriam mais elevados. Além disso, essa população parecia não ter correlação entre os valores de EIMC e os níveis séricos de endocan. Ao avaliar os grupos de pacientes pré-diabéticos e normoglicêmicos, houve correlação entre EIMC e endocan sérico em pacientes normoglicêmicos, mas não em pacientes pré-diabéticos.

Assim, os níveis séricos de endocan seriam baixos em pacientes pré-diabéticos. Esse resultado provavelmente ocorreria devido ao estado hiperinsulinêmico dos pacientes. É difícil distinguir o efeito da resistência à insulina da hiperinsulinemia compensatória. ^1^ A sinalização da hiperinsulinemia atuaria na regulação da produção de óxido nítrico. ^11 , 12^ Além de seu papel nos mecanismos ateroscleróticos, atenuaria a resposta inflamatória sistêmica induzida por endotoxinas, diminuindo a expressão de TNF-α e aumentando a cascata anti-inflamatória. ^13 , 14^ O efeito redutor da hiperinsulinemia sobre os níveis de TNF-α explicaria a diminuição dos níveis séricos de endocan, que são secretados pelo TNF-α e a interleucina-1 beta (IL-1β). ^15^

Em resumo, o estudo mostrou que os níveis de endocan diminuíram em pacientes pré-diabéticos, e esse resultado provavelmente estaria relacionado à hiperinsulinemia nessa população. No entanto, apesar dos níveis séricos de endocan serem normais, a aterosclerose subclínica no grupo de pacientes não pode ser descartada, uma vez que essa mesma população apresentou alterações no EIMC. Além disso, o fato de os valores do índice de massa corporal serem semelhantes entre os dois grupos avaliados pode ter contribuído para o achado acima.

No entanto, os autores reconhecem algumas limitações: o pequeno número de pacientes incluídos, o trabalho foi realizado em um único centro e o desconhecimento do momento em que esses pacientes estariam na fase pré-diabética pode ter influenciado nos resultados. Eu acrescentaria a esta lista a dosagem de CRPhs. Talvez este marcador possa acrescentar alguma informação ao cenário do estudo.

Estudos mais robustos em populações mais diversificadas seriam necessários para entender melhor esse biomarcador.

Por enquanto, o uso do endocan proteoglicano na estratificação do risco cardiovascular para pacientes em prevenção primária, mas de risco alto e intermediário, pode explorar essa lacuna ainda não totalmente compreendida no endotélio arterial. Se assim for, evoluiremos cada vez mais nas fronteiras da medicina translacional.

Os autores devem ser parabenizados por tomarem a iniciativa de explorar um assunto tão conflitante!

## References

[1] Arman Y, Atici A, Altun O, Sarikaya R, Yoldemir A, Akarsu M (2022). Can the Serum Endocan Level Be Used as a Biomarker to Predict Subclinical Atherosclerosis in Patients with Prediabetes?. Arq Bras Cardiol.

[2] Park C, Guallar E, Linton JA, Lee DC, Jang Y, Son DK (2013). Fasting Glucose Level and the Risk of Incident Atherosclerotic Cardiovascular Diseases. Diabetes Care.

[3] Stein JH, Korcarz CE, Hurst RT, Lonn E, Kendall CB, Mohler ER (2008). Use of Carotid Ultrasound to Identify Subclinical Vascular Disease and Evaluate Cardiovascular Disease Risk: A Consensus Statement from the American Society of Echocardiography Carotid Intima-Media Thickness Task Force. Endorsed by the Society for Vascular Medicine. J Am Soc Echocardiogr.

[4] Santos IS, Goulart AC, Brunoni AR, Kemp AH, Lotufo PA, Bensenor IM (2015). Anxiety and Depressive Symptoms are Associated with Higher Carotid Intima-Media Thickness. Cross-Sectional Analysis from ELSA-Brasil Baseline Data. Atherosclerosis.

[5] Webb DR, Davies MJ, Gray LJ, Abrams KR, Srinivasan B, Das S (2010). Searching for the Right Outcome? A Systematic Review and Meta-Analysis of Controlled Trials Using Carotid Intima-Media Thickness or Pulse Wave Velocity to Infer Antiatherogenic Properties of Thiazolidinediones. Diabetes Obes Metab.

[6] Lorenz MW, Markus HS, Bots ML, Rosvall M, Sitzer M (2007). Prediction of Clinical Cardiovascular Events with Carotid Intima-Media Thickness: A Systematic Review and Meta-Analysis. Circulation.

[7] Klisic A, Kavaric N, Vujcic S, Mihajlovic M, Zeljkovic A, Ivanisevic J (2020). Inverse Association between Serum Endocan Levels and Small LDL and HDL Particles in Patients with Type 2 Diabetes Mellitus. Eur Rev Med Pharmacol S Sci.

[8] Lv Y, Zhang Y, Shi W, Liu J, Li Y, Zhou Z (2017). The Association between Endocan Levels and Subclinical Atherosclerosis in Patients With Type 2 Diabetes Mellitus. Am J Med Sci.

[9] Belongie KJ, Ferrannini E, Johnson K, Andrade-Gordon P, Hansen MK, Petrie JR (2017). Identification of Novel Biomarkers to Monitor β-cell Function and Enable Early Detection of Type 2 Diabetes Risk. PLoS One.

[10] Klisic A, Kavaric N, Stanisic V, Vujcic S, Spasojevic-Kalimanovska V, Ninic A (2019). Endocan and a Novel Score for Dyslipidemia, Oxidative Stress and Inflammation (DOI score) are Independently Correlated with Glycated Hemoglobin (HbA1c) in Patients with Prediabetes and Type 2 Diabetes. Arch Med Sci.

[11] Steinberg HO, Brechtel G, Johnson A, Fineberg N, Baron AD (1994). Insulin-Mediated Skeletal Muscle Vasodilation is Nitric Oxide Dependent. A Novel Action of Insulin to Increase Nitric Oxide Release. J Clin Invest.

[12] Montagnani M, Chen H, Barr VA, Quon MJ (2001). Insulin-Stimulated Activation of eNOS is Independent of Ca2+ but Requires Phosphorylation by Akt at Ser(1179). J Biol Chem.

[13] Jeschke MG, Klein D, Bolder U, Einspanier R (2004). Insulin Attenuates the Systemic Inflammatory Response in Endotoxemic rats. Endocrinology.

[14] Brix-Christensen V, Andersen SK, Andersen R, Mengel A, Dyhr T, Andersen NT (2004). Acute Hyperinsulinemia Restrains Endotoxin-Induced Systemic Inflammatory Response: An Experimental Study in a Porcine Model. Anesthesiology.

[15] Lassalle P, Molet S, Janin A, Heyden JV, Tavernier J, Fiers W (1996). ESM-1 is a Novel Human Endothelial Cell-Specific Molecule Expressed in Lung and Regulated by Cytokines. J Biol Chem.

