# Supplementary material for: Biomarcadores Endoteliais e Medicina Translacional: Ainda um Desafio
Source: Arq Bras Cardiol. 2022 Oct 5;119(4):551–2. [Article in Portuguese] doi: 10.36660/abc.20220533 (PMC9563896; doi:10.36660/abc.20220533)
Supplement: Translation

# Endothelial Biomarkers and Translational Medicine: Still a Challenge

**Authors:** Renato Jorge Alves

**Affiliations:** 1 Brazil Irmandade da Santa Casa de Misericórdia de São Paulo – Departamento de Medicina, São Paulo, SP – Brazil; 2 Brazil Faculdade de Ciências Médicas da Santa Casa de São Paulo, São Paulo, SP – Brazil

**Keywords:** Atherosclerosis, Prediabetic State, Insulin Resistance, Diabetes Mellitus, Carotid Intima-Media Thickness, Obesity

The article “Can the Serum Endocan Level Be Used as a Biomarker to Predict Subclinical Atherosclerosis in Patients with Prediabetes?”^1^ addressed a topic of the greatest relevance and originality, the diagnostic investigation of subclinical atherosclerosis in pre-diabetic patients. Therefore, the study evaluated the endothelial cell-specific molecule-1 (ESM-1) endocan concentration in pre-diabetic patients to verify the role of serum endocan levels in detecting subclinical atherosclerosis, aided by measuring the intima-media layer of the carotid arteries thickness (IMT).

Due to the global epidemic of obesity, there is an increasing number of patients diagnosed with type 2 diabetes. However, preceding this morbidity, an even greater contingent of individuals is hidden, those in the pre-diabetic or insulin resistance phase.

It is known that insulin resistance increases cardiovascular risk, as it is related to a worse lipid profile, pro-inflammatory state, and endothelial dysfunction. Expressive endothelial changes occur, increasing from the expression of inflammatory markers.^2^

On the other hand, adding tools that contribute to the early detection of these biomarkers in this population is essential to try to change the disease evolution. Among them, the IMT measurement has become one of the main assessment and diagnosis methods. Moreover, in addition to being an easy-to-perform test, it directly correlates with early endothelial alteration in atherosclerotic disease.^3 - 6^

Endocan, in turn, is a proteoglycan released by endothelial cells from inflammatory cytokines, which would regulate the inflammatory process. It is closely linked to endothelial injury. It has been shown that serum endocan levels would be higher in diabetic patients and acute coronary syndrome.^7 , 8^

However, intriguing results indicated that the serum levels of endocan would be low or unchanged in pre-diabetic patients,^9 , 10^ in contrast to the IMT values, which would be higher. In addition, this population appeared to have no correlation between IMT values and serum endocan levels. When evaluating pre-diabetic and normoglycemic groups of patients, there was a correlation between IMT and serum endocan in normoglycemic patients but not in pre-diabetic patients.

Thus, serum endocan levels would be low in pre-diabetic patients. This result would probably occur due to the hyperinsulinemic state of the patients. It is difficult to distinguish the effect of insulin resistance from compensatory hyperinsulinemia.^1^ Hyperinsulinemia signaling would act in the regulation of nitric oxide production.^11 , 12^ In addition to its role in atherosclerotic mechanisms, it would attenuate the systemic inflammatory response induced by endotoxins, decreasing the expression of TNF-α and increasing the anti-inflammatory cascade.^13 , 14^ The reducing effect of hyperinsulinemia on TNF-α levels would explain the decrease in serum endocan levels, which are secreted by TNF-α and interleukin-1 beta (IL-1β).^15^

In summary, the study showed that endocan levels decreased in pre-diabetic patients, and this result would likely be related to hyperinsulinemia in this population. However, despite serum endocan levels being normal, subclinical atherosclerosis in the group of patients cannot be ruled out since this same population presented changes in IMT. Furthermore, the fact that the body mass index values were similar between the two groups evaluated could have contributed to the above finding.

However, the authors recognize some limitations: the small number of included patients, the work was carried out in a single center, and the lack of knowledge of the time when these patients would be in the pre-diabetic phase could have influenced the results. I would add to this list the dosage of CRPhs. Perhaps this marker could add some information to the study setting.

More robust studies in more diversified populations would be necessary to understand this biomarker better.

For now, the use of endocan proteoglycan in the stratification of cardiovascular risk for patients undergoing primary prevention but at high and intermediate risk may explore this gap not yet fully understood in the arterial endothelium. If so, we will increasingly evolve on the frontiers of translational medicine.

The authors should be congratulated for taking the initiative to explore such a conflicting subject!
